# A protocol for the methodological steps used to evaluate the alignment of rehabilitation services in the Western Cape, South Africa with the National Rehabilitation Policy

**DOI:** 10.1186/s12913-017-2141-3

**Published:** 2017-03-14

**Authors:** Gubela Mji, Anthea Rhoda, Sue Statham, Conran Joseph

**Affiliations:** 1 Department of Neurobiology, Care Sciences and Society, Karolinska Institutet, Stockholm, Sweden; 2 Physiotherapy Department, University of the Western Cape, Cape Town, South Africa; 3 Centre for Rehabilitation Studies, Medicine and Health Science Faculty, Stellenbosch University, Stellenbosch, South Africa; 4 Physiotherapy Department, Stellenbosch University, Stellenbosch, South Africa

**Keywords:** National Rehabilitation Policy of South Africa (NRP), Evaluation of rehabilitation policy, Rights based approach to rehabilitation, Community based rehabilitation (CBR), United Nations Convention for Persons with Disabilities (UNCRPD), International Classification for Functioning Health and Disability (ICF), Kaplan’s Framework for Organisational Capacity, Realistic evaluation research

## Abstract

**Background:**

Rehabilitation medicine plays an integral part in attainment of optimal functioning after injury or disease. The National Rehabilitation Policy of South Africa (NRP) (2000) highlights the need for access to professional health care services, redistribution and optimal utilisation of resources and research in the field of disability and rehabilitation. The government further ratified the Convention on the Rights of Persons with Disabilities (CRPD) (2007), which validate the urgency in advancing the agenda of persons with disabilities. This paper outlines the methodological plan for evaluating rehabilitation services in the Western Cape, South Africa against the aims and objectives of the NRP as well as its principles and concepts. The evaluation process further focused on specific articles in the CRPD that were aligned with disability, health and rehabilitation.

**Methods/Design:**

A mixed-method design was used to evaluate the alignment of rehabilitation services with the NRP in the Western Cape. Four rehabilitation study settings were selected to ensure that both inpatient and outpatient rehabilitation levels of care were covered at different contexts (rural and peri-urban). The sites were checked for the most prevalent rehabilitation-related conditions to ensure the identification of suitable instruments for measuring rehabilitation outcomes. Each study setting was linked to two researchers with one exploring the rehabilitation organizational structure of the sites and the other exploring the client outcomes after receiving rehabilitation services. Patients were evaluated at baseline and discharge, within seven days after admission and seven days prior to discharge. The evaluation was based on the rehabilitation organizational capacity to provide patient-oriented rehabilitation and the measurement of rehabilitation outcomes. Kaplan’s framework of organisational capacity was used in the context of each study setting. For the measurement of service users’ outcomes, the International Classification of Functioning, Disability and Health was used (ICF). Standardised outcome measures were adopted for the domains of impairment, activity and participation. The World Health Organisation Community-Based Rehabilitation guidelines were used as guiding principles and concepts as suggested in the NRP.

**Discussion:**

This is a groundbreaking methodological exploration that offers both study methods and instruments to measure rehabilitation services at both in-patient and out-patient rehabilitation services.

## Background

The aim of this paper is to present the protocol for the process of developing methodological steps for the realistic evaluation [1] of institution-based inpatient and outpatient rehabilitation services in four sites in the Western Cape. This evaluation was based on the alignment of rehabilitation services in four sites, at different levels of care provision, in the Western Cape with the National Rehabilitation Policy (NRP) of 2000 [2]. The National Rehabilitation Policy (NRP) was adopted in 2000 and the perception of the rehabilitation professionals is that little attention and resources have been given to move rehabilitation services from policy to practice [3]. It is not clear what plans have been put in place by both the National and provincial governments to implement this policy [4]. The general lack of research evidence regarding the effect of institution-based rehabilitation and its effect on the quality of life of clients could lead to a lack of proper planning and resourcing of rehabilitation services [5]. These institution based rehabilitation services had never been measured against the seven objectives of the NRP, despite the fact that one of the objectives is directed towards initiatives in rehabilitation and development of evaluation strategies for rehabilitation programs [2]. This has a negative impact on resource allocation from government policy implementers [4]. Different areas in the Western Cape developed different models for service delivery; thus, these models need to be measured against what is envisaged in the NRP – it will be important to ascertain the gaps between policy and implementation [6].

The evaluation of service delivery systems is listed by Mitchel [7] as a focus research area in rehabilitation and has been described as a systemic and continuous process of information provision for the purpose of determining the value of a programme. The aim of the methodological steps was to outline an evaluation process to compare the situation in a given programme to agreed standards and to programme objectives [2]. A solid base for planning and management, systematic information gathering is needed about: (a) the status of individual clients, (b) the services offered, (c) the programme environment, and (d) the relationships between the clients, the programme and the programme environment [8]. Thus, the core of this paper was to explore the steps that could be taken to develop a protocol to investigate the capacity and mechanisms used on the four sites to deliver comprehensive institutional based rehabilitation services and the outcomes and benefits of these services to clients using these services. This project was funded by the South Africa Netherlands Research Programme on Alternative development (SANPAD) (Project PO8/16: 2009–2012).

### A brief background and synopsis of the National rehabilitation policy (NRP)

The reforms of 1994 in South Africa necessitated adjustments of existing health policies and the development of new ones. The overarching aim was to improve access to health services especially to the poor. Primary health care was identified as the vehicle to deliver health services. Rehabilitation is the third pillar of the primary health care service model [9]. It is against this backdrop that the National Rehabilitation Policy (NRP) was developed and published in 2000 [2].

According to the World Health Organisation, rehabilitation aims to enable persons with disabilities to reach and maintain their optimal functional levels by providing them with the tools they need to attain independence and self-determination [10]. To achieve the goal of rehabilitation that has been stated by WHO, the NRP (2000) has stated seven 7 objectives that underpin rehabilitation services. The seven 7 objectives of the NRP are presented below:

- To improve accessibility of rehabilitation services
- To establish mechanisms for intersectoral collaboration in order to implement a comprehensive rehabilitation programme.
- To facilitate appropriate allocation of resources, and encourage their optimal utilisation.
- To facilitate human resource development that takes into account the needs of both the service providers and the consumers.
- To encourage the development and implementation of monitoring and evaluation strategies for rehabilitation programmes.
- To ensure participation of persons with disabilities in planning, implementation and monitoring of rehabilitation programmes.
- To encourage research initiatives in rehabilitation and related areas (1; p.2).

There is a need to evaluate the effects of these seven objectives on clients who have attended institution-based rehabilitation services in the Western Cape. Service delivery models that use the medical model of rehabilitation are rejected by disabled people’s organizations because of the emphasis on adjusting the person to the existing systems and norms through – mainly – medical and therapeutic interventions [11]. In spite of current emphasis on the social model by the disability activists, medical and rehabilitation services remain important as a gate way for equal opportunity and full participation of disabled people [12]. The rendering of comprehensive rehabilitation services is a constitutional right of disabled people in South Africa [13]. The NRP mentions clearly the need for protection of human rights of persons with disabilities. It further states that these principles are based on the premise that “rehabilitation includes not only the training of disabled people, but also interventions in the general system of society, allowing for adaptations of environment and protection of human rights [11]”. To demonstrate its alignment with human rights, the NRP includes the principles of equality; social justice and equity, solidarity, dignity and integration and participation.

**Fig. 1:**
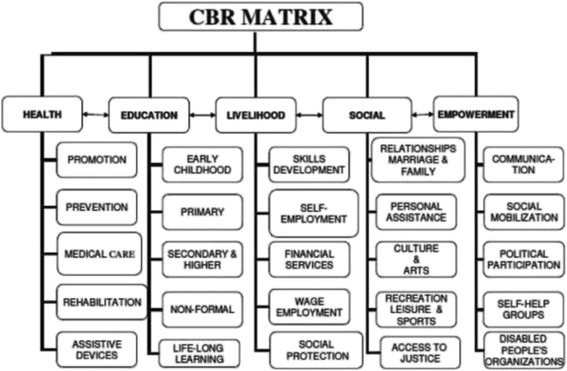
CBR Matrix (WHO 2010)

As a practical means of adopting a rights-based approach to rehabilitation services and equalization of opportunities for disabled people, the NRP has adopted Community Based Rehabilitation (CBR) strategy and describes it as an approach to delivering services and not the service itself [2]. At the core of CBR is the notion of empowerment and full community integration and participation of the people with disabilities [14]. Thus rehabilitation at all four of the selected sites should thus function within the framework of CBR (Table 1, Fig. 1). Velema and Cornielje [3] highlighted the need for baseline data as well as well-developed and well implemented information systems in CBR. This evaluation model intends to evaluate the CBR component of the four sites in the Western Cape thus assisting with an instrument that could be validated in other CBR programmes in Africa.

**Table 1:** Principles of CBR (WHO 2010)

CBR principles	Definition
Participation	Active contribution of people with disabilities in CBR practice and monitoring.
Inclusion	Placing people with disabilities and their issues in the mainstream.
Sustainability	Benefits of the programme must be lasting beyond life of programme.
Self-advocacy	Consistent involvement of service users in terms of defining practice and indicators.

The recently ratified United Nations Convention on the Rights of Persons with Disability (UNCRPD) is the latest instrument that raises issues of equity for persons with disability. It calls for governments to mainstream disability in policies of their countries. South Africa is the first African country that ratified the UNCRPD. Rehabilitation features strongly within the UN Convention with articles 9, 19, 20, 25 & 26 giving clear indicators regarding how to respond to the rehabilitation needs of the disabled people. These articles resonate in the objectives of the NRP [15]. This evaluation research, in addition to investigating the alignment of the rehabilitation services to the objectives of the NRP will further examine how these services are responding to the 5 select articles (Table 2) of the UNCRPD. Results from this will assist the South African government to identify gaps within its rehabilitation services and the UNCRPD. Using the UNCRPD as a benchmark will elevate this research to a more internationally generalisable level [15].

**Table 2:** Selected articles on rehabilitation from UN convention for the rights of persons with disabilities

Article no.	Topic area
9	Accessibility
19	Living independently and being included in the community
20	Personal mobility
25	Health
26	Habilitation and Rehabilitation

### The development of a collaborative research group

A small team of three researchers started planning an evaluation of one site in the Western Cape. The approach was to demonstrate the effect of rehabilitation with the aim of convincing the provincial government of the need for allocation of resources for rehabilitation service in this site. It was during this period of proposal writing for this one site that a wider call came from SANPAD inviting people to send proposals for a collaborative research study. The Centre for Rehabilitation Studies offers advanced interdisciplinary postgraduate rehabilitation studies. It is a platform for the disability and rehabilitation debate and is in partnership with the Provincial Government and Stellenbosch University to ensure evidence-based input into the programs and policies of the Province. Hence, it was in excellent position to coordinate this collaborative research. Initially, a comprehensive team of approximately 20 people joined the research team and developed a reference group for the project. The team was a mixture of young and more mature rehabilitation professionals from public institution-based rehabilitation services and academic departments (SU: CRS, Physiotherapy and Occupational Therapy, UWC: Physiotherapy Department and rehabilitation professionals from provincial health department). The proposal was accepted and funded by SANPAD that allowed the reference group the opportunity to implement the evaluation plan of the four sites.

### The development of the hypotheses for the study

- Firstly, we wanted to evaluate whether the NRP with its seven objectives is aligned with the rehabilitation services at the four sites in the Western Cape.
- Secondly, we wanted to measure whether rehabilitation services as an organizational structure in the four sites can deliver comprehensive rehabilitation services.
- Thirdly, we wanted to measure, using rehabilitation outcome measures, whether the patients that enter the four study settings do receive comprehensive rehabilitation.
- Lastly we wanted to align our findings with the 7 objectives of the NRP and do a gap analysis with regard to whether the NRP has lived to its promises.

## Methods

### The selection of the four rehabilitation research sites

Rehabilitation services in the Western Cape are subject to several national and provincial policies. The rehabilitation of physically disabled persons in the Western Cape takes place at various levels of health care and at a variety of institutions. This realistic evaluation research will critically analyze services rendered at four sites within the public sector. The context, mechanisms and outcomes (CMO) for each site were investigated. The sites were purposively selected as each site represents a different level of intervention and serves a different population. The Western Cape Rehabilitation Centre (WCRC) is a specialized in-patient rehabilitation centre that offers both in and out-patient services. In patient referrals come from the Western, Eastern and Northern Cape, while out-patient therapy is limited to patients in the direct vicinity of the Centre. For inpatient rehabilitation, the WCRC admits annually approximately 1160 clients with mainly neurological health conditions. The five most prevalent health conditions constitute about 94% of all clients admitted to the centre (Table 3). The Community Health Centres (CHCs) deliver Primary Health Care services to the urban and peri-urban communities in their direct vicinity. Therapists, who often work in isolation, provide acute and rehabilitation services in these CHCs. The Bishop Lavis (BL) CHC has a multidisciplinary rehabilitation team which is run by Stellenbosch University with support from the Provincial Health Department. At BL CHC patients are treated on an individual basis, where between 59 and 154 per month are seen for occupational therapy services, 65–325 for physiotherapy services, and 20–120 patients for speech therapy. Guguletu CHC has full time physiotherapy services that manage between 4500 and 5000 patients a year and a part time occupational service which sees between 350 and 400 patients a year. Elangeni Out-patient Rehabilitation Centre also offers multidisciplinary rehabilitation and serves the rural community. Infrastructure and resource limitations as well as the vast area to be serviced pose particular challenges for team work and an interdisciplinary approach to rehabilitation in this area.

**Table 3:** Most common conditions identified from the rehabilitation services in the study sites

Health conditions	WCRC^a^	Gugulethu CHC^a^	Bishop Lavis	Elangeni CHC^a^
Spinal Cord Injury	X			
Stroke	X	X	X	X
Amputations	X			
Head Injuries	X			
Peripheral Neuropathy	X			
Back and neck pain		X	X	X
Upper limb injuries		X	X	X
Lower limb injuries		X	X	X
Arthritis			X	

^a^ *WCRC* Western Cape Rehabilitation Centre, *CHC* Community Health Centre

### The development of the conceptual framework for the methodological steps used to evaluate rehabilitation services in four sites in the Western Cape

A series of steps were developed using aspects of the realistic approach to evaluation research and taking into account the concept of the context of the rehabilitation services and the rehabilitation outcomes of the clients receiving these services [1, 16]. Mixed research methodologies were used focusing on the seven objectives of the NRP and the ability of the four sites to deliver what these objectives promised. This mixed research methods approach included Kaplan’s framework of developing organizational capacity to evaluate the delivery of effective services, while the ICF framework was used to measure key outcomes of clients receiving rehabilitation from the selected sites.

Continuous discussions were held by the research group to develop a unified conceptual framework for the evaluation study. Discussions concentrated on:

- The capacity of the four sites as organizational structures to deliver rehabilitation services
- And the rehabilitation outcomes of the patients receiving the services as well as client satisfaction.

**Fig. 2:**
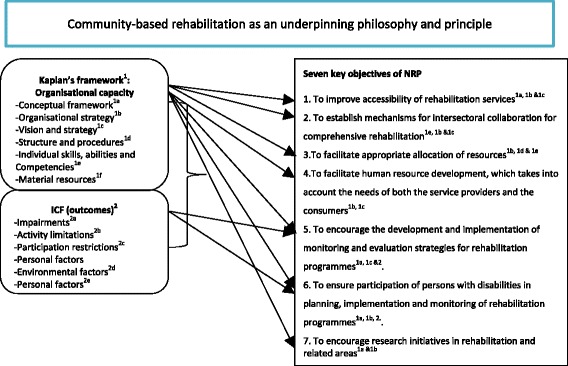
Integration and alignment of conceptual frameworks to evaluate rehabilitation services in the Western Cape, South Africa. The superscript legends under key objectives of the NRP corroborate with the level or domain of the conceptual framework that is aligned to the evaluation of each objective: Kaplan’s framework = ^1^, Conceptual framework = ^1a^, Organisational strategy = ^1b^, Vision and strategy = ^1c^, Structure and procedures = ^1d^, −Individual skills, abilities and Competencies^1e^, Material resources^1f^: ICF outcomes = ^2^, −Impairments^2a^ , Activity limitations^2b^, Participation restrictions^2c^ , Environmental factors^2d^ , Personal factors^2e^. The *arrows* demonstrate only examples of the alignment of the framework towards the evaluation of objectives

It has already been specified in the discussion of the NRP that the NRP has seven objectives and links to CBR and the UNCRPD. We concluded that these will be used as yardsticks to evaluate the ability of the rehabilitation services in the four sites to deliver rehabilitation services. On one hand we decided to develop indicators based on the seven objectives of the NRP, aspects from the CBR matrix and 5 health and rehabilitation articles from the UNCRPD. We saw that the fulfillment of these indicators by the four sites would give us the capacity to deliver rehabilitation services based on the organizational framework by Kaplan [16]. Validated rehabilitation outcome measures underpinned by the domains of the ICF were selected to assess clients before and after receiving rehabilitation services. Each site was investigated in context (i.e. the most prevalent conditions, demographics etc.) with the Kaplan model used to investigate the mechanisms used at each setting and the outcomes of the patients were measured using questionnaires and standardized outcomes measures Fig. 2.

### The use of Kaplan’s framework for organizational capacity as a method of evaluating the capacity of the four sites to deliver rehabilitation services

**Fig. 3:**
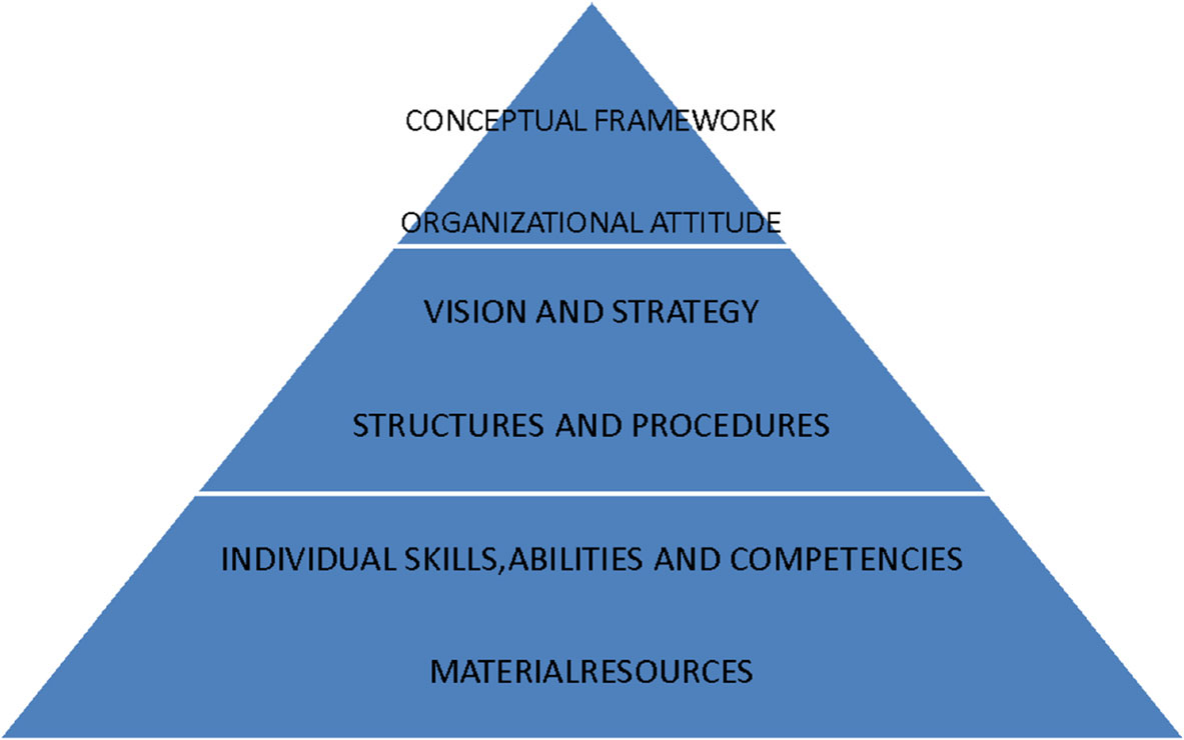
Kaplan’s conceptual framework of an organizational capacity

When looking at the ability of the four sites to deliver rehabilitation services to clients, we decided to use Kaplan’s model of evaluating organizational capacity [16]. Kaplan derived at a criteria consisting of number of elements that must be present for any organization to be effective. According to Kaplan, there must be a conceptual framework, which reflects the organization’s understanding of the world. The organization must have an organizational attitude which includes an acceptance of responsibility for surrounding conditions and the confidence to act in a way which it feels will be effective. There must be a clear organizational vision and strategy with a sense of purpose and will. The organization must have structures and procedures which reflect and support the vision and strategy. Relevant individual skills and abilities must be available as well as sufficient and appropriate material resources. All these elements that Kaplan described would be targeting the needs of disabled people and their service providers. Figure 3 illustrates Kaplan’s conceptual framework of an organizational capacity.

At the bottom of the hierarchy are quantifiable and measurable elements of capacity which can be worked with. The elements nearer the top of the hierarchy are not easily assessed and are observable only through the effects they have and they largely determine the organizational capacity. The framework describes the elements of capacity but it cannot predict or determine change processes. When tensions arise between the needs of the health care institution and those of the team and the client, a conceptual framework can help to retain the client focus [17]. In this study, Kaplan framework was used to look at the capacity of the centers in the 4 sites to deliver an institutional-based rehabilitation service.

The NRP was seen as having promised certain deliverables through its objectives and principles, hence key indicators were drawn from the objectives of the National Rehabilitation Policy, the 5 relevant articles of the UNCRPD and the Community Based rehabilitation model, and we finally determined how these indicators were aligned with the 6 elements of organizational capacity named by Kaplan. Questionnaires were developed from these indicators for clients, service providers and managers to evaluate the service delivery of the four sites. A description of the methodological steps used to evaluate the alignment of the service provision of the four sites in the western Province with the NRP.

### The evaluation of the capacity of the four sites to deliver rehabilitation services (organizational capacity – underpinned by Kaplan framework)

The research team searched for instruments to be used in this study; however, no suitable questionnaires were found which could be used to ask relevant questions to the service providers and the managers. Various questionnaires were found which addressed some of the issues that needed to be interrogated but there were none that were able to interrogate all the objectives of the National Rehabilitation Policy, the selected articles of the UNCRPD, the CBR principles and the ICF. Also during the questionnaire design, we utilized some of the primary health care criteria for the delivery of comprehensive primary health care, for example accessibility, acceptability, affordability and equity. Therefore the research team designed three questionnaires, one for service providers, one for the manager of the centre and one for the manager of the non-governmental organization.

The research team of the SANPAD funded study was tasked to do an in depth analysis of the objectives of the National Rehabilitation Policy, the relevant five articles of the United Nations Convention on the rights of Persons with disabilities, the Kaplan Organizational Model, Community Based Rehabilitation, the International Classification of Function and Disability framework and key Human Rights Documents. All documentation was analysed by the group and all possible questions drawn up to interrogate each of the seven objectives of the National Rehabilitation Policy. Next, the five relevant articles of the UNCPRD were analysed and questions drawn up relating to these five articles. These questions were then added to the original questions. The group checked that the objectives of Community Based Rehabilitation were also included as well as Human Rights issues.

A spreadsheet was drawn up with indicators and the questions relating to each indicator. The relationship between each question and the five elements of the Kaplan Model was then determined. Three key informant groups were then decided upon. These four groups were the service providers, the manager of the Community Health Centre and a Non-governmental organization manager and persons with disabilities who were the consumers of the services. Questions relating to catchment area as well as the rehabilitation human resource provision were answered by the facility managers. Persons with disabilities participated in responding to questions related to participation of persons with disabilities and health service-related questions. We also explored issues on the structures and procedures and those related to guidelines on clinical treatment, availability and training of staff, procurement of resources as well as administration-related questions. For some questions, there were more than one key informant. Draft questionnaires were then drawn for the key informant groups. The fourth group of key informants was the rehabilitation service clients and standardised questionnaires were used that answered the relevant questions. The standardised questionnaire was mainly based on the ICF checklist for environmental factors. Once the questionnaires had been piloted and the adjustments made, they were sent to an expert in managing rehabilitation services and an expert in rehabilitation research for validation purposes. Minor changes were indicated, and suggestions were integrated to reflect the intended content.

### The use of the International Classification of Function, Disability and Health (ICF) to determine the rehabilitation outcomes

**Fig. 4:**
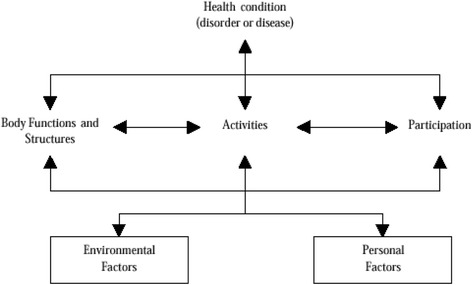
International Classification of Functioning, Disability and Health - ICF [18]

Moving from capacity of institutions rendering rehabilitation services to outcomes, the ICF was used to assist in the understanding of the complex interaction between features of the biological, psychological, cultural and social factors of disability [18]. In this model, (see Fig. 4), which is framed within the biopsychosocial perspective model, disability is defined as the South African ratified UNCRPD outcome of the interaction between a person’s health condition and the context in which the person finds themselves [19]. The three classifications of experiences of physical and functional impairments involving an organ or body part; activity limitation involving the whole body of a person towards achieving a certain functionality and participation restriction involving the person interacting with the environment are at the core of the classification [18]. This classification shifts conceptualizing disability and health within a continuum whereby certain variables have to be altered to ultimately have a direct impact on the status of one’s health. This context includes external environmental factors (e.g. assistive devices, physical accessibility, societal attitudes), and those factors internal to the person (e.g. age, sex, coping skills, personality). As elements of the body and personal and external environmental factors change, so the outcome will also change.

As well as recognising the impact of individual bodies and social factors in understanding disability, the main principle of the biopsychosocial model is to understand disability as a continuum. In analysing this notion of rehabilitation critically, it can be seen that using the ICF incorporates both the medical and social models in rehabilitation. For example, rehabilitation inputs can be focused directly on activity and participation, or might target body function issues (i.e. contractures, pressure sores) [19, 20]. This is similar to what the NRP of 2000 through its 7 objectives promises to deliver to disabled people. The ICF was used as an overarching conceptual understanding of clients receiving rehabilitation services. We used rehabilitation outcome measures to determine the performance of a client receiving rehabilitation services at the different levels of the ICF. The overall aim of rehabilitation, according to the NRP, is the attainment of social participation and integration into society. Limited information is available concerning the operational meaning of participation, social participation and integration. This notion is evident in the literature as well. We were in a prime position to evaluate the tacit meaning of social participation and integration from the perspective of the service providers (using Kaplan’s framework) and the service users, using the ICF.

### The use of outcome measures to evaluate recipients of rehabilitation interventions in the four sites

#### Process followed

The process started with the identification of the five most prevalent conditions at each site (Table 3). To be in line with what the ICF suggestion of starting with the impairment, it was decided that conditions such as backache would also be included as it often impacted on the level of activity and participations of individuals. After identifying the conditions that would be included in the different settings, a literature review was conducted to identify which instruments would be best suited to measure the outcomes at the level of impairment, activity and participation for each condition. Factors that were considered when reviewing which instruments could be used in the study included; psychometric properties such as validity, reliability and responsiveness [21], but also factors concerning translations, cross-cultural adaptations and the user-friendliness of the tool, owing to the fact that a large part of the clients do either do not or only have basic education [22–24]. With these hindering factors taken into account, we scrutinized the literature for appropriate measures.

Following the review, it was found that no suitable instrument was available to measure impairments and participations restrictions related to each of the health conditions. A condition-specific instrument was therefore developed to measure impairments, standardized measures were used to collect data concerning activity limitations of the five conditions at each site, and a previously used generic instrument was adapted and validated to collect participation data. In addition three questionnaires were developed to collect contextual information. The development of these instruments is presented below. The study on the rationale, design and pilot of the impairment outcome measure is in progress.

##### Impairment measures

Following a focus group discussion of therapists, researchers and academics involved in this project, a condition-specific impairment measure for each health condition was developed. For the development process, all primary impairments related to the health condition, given that it is routinely assessed in practice, were identified. The impairment variables were identified via the aforementioned study group consisting of therapists, researchers and academics, the ICF framework on body structures and functions [18], the ICF core sets for each health condition and for each management period, and the literature [25–27].

A data extraction sheet was developed and distributed to experts in the field of rehabilitation with the aim of ensuring the face and content validity. All questions and recommendations from the expert panel were considered and integrated, with the final version approved by them. In addition, the inter-rater reliability of the impairment extraction sheet was conducted on the medical records of 20 patients. Two reviewers independently captured the data from the 20 patient records. The data were captured and analysed using Statistical Package for Social Sciences version 18. The Intraclass Correlation Coefficient (ICC) of all the items averaged together for both raters ranged between 0.471 and 1, which indicates moderate to perfect agreement [28].

##### Instruments used to measure activity

A number of suitable outcome measures were available to measure activity limitations of all health conditions covered in this study. All the standardised measures decided on presented with good psychometric properties and translated versions were available for most. In cases where translated versions were not readily available, we initiated the process and we are currently busy with its validation. The Barthel Index was used to assess activity limitations in persons with stroke, amputations and traumatic brain injury, whereas the spinal cord independence measure III was used among those living with a spinal cord injury, and the Screening Activity Limitation and Safety Awareness outcome measure was used among persons with peripheral neuropathy. For back pain, we used both the Oswestry ODI version 2.0 and the Clinical Mobility Scale. For persons with arthritis and hands/upper limb injuries, we used the Arthritis Impact Measuring Scale and the Disability of the Arm Shoulder and Hand, respectively [29–31].

##### Participation and health-related quality of life measure

Since limited participation measures are available, especially generic outcome measures, the Zambian survey on living conditions that was utilised to study the influence of physical disabilities on activity and participation of patients in Zambia [32] was adapted for the purpose of our study. The Zambian questionnaire on living conditions consists of nine 9 sections that measure constructs of impairment, activity limitation and participation restriction within the ICF framework. The nine constructs relate to functioning of the senses, communication, mobility, caring for self, household life, interpersonal behaviour, important areas of life and community, social and civic life. The items relating to impairments were removed as those aspects were extracted from the medical folders. Each item contained in the measure is described in terms of the relative ease or difficulty one experiences with the execution of the task or action, with it being scored on a scale ranging from 0–4 (0 = no problem; 4 = inability to perform the activity). The adapted questionnaire was reviewed by the reference group for content and face validity, and a test-retest reliability pilot study was conducted. For the reliability test, ten chronic stroke patients who were part of a stroke cohort were asked to participate. The participants were interviewed at one point and two week later using the questionnaire. The information for activity was analysed separate from that for participation. The ICC scores for the participation domains ranged from .431 to 1 and that for activity ranged from .410 to 1.0, indicating acceptable agreement between testing occasions.

Concerning health-related quality of life, the EQ-5D was used across all participating centres and health conditions. This measure consists of five items that are thought to best represent an individual’s quality of life in the presence of a disabling health condition. The EQ-5D has been widely used in South Africa for the valuation of quality of life, and the measure presents with adequate psychometric properties, as tested in the local context [33] Table 4.

**Table 4:** Classification of outcome measures using the International Classification of Functioning, Disability and Health (ICF)

		ICF				
Instruments	Domains	Personal factors	Environmental factors	Body function	Activity	Participation
Self-developed questionnaire						
Personal characteristics	- Age	X				
	- Gender	X				
	- Marital status	X				
	- Educational level	X				
	- Ethnicity	X				
Health status	- Time since health event	X				
	- Recurrence of health event	X				
	- Co-morbidity	X				
	- Assistive gait devices	X				
	-Living conditions and basic needs	X				
Environmental factors questionnaire	- Access to health facility -Environmental hazards -Satisfaction of care		X X			
Self-report questionnaires						
Impairment data gathering sheet	- Severity of health event -Documented impairments			X		
Activity measure,	- Functional ability				X	
Zambian questionnaire	- Functional abilities and participation				X	X
EQ-5D (Quality of life)	- Health-related quality of life					X

*ICF* International Classification of Functioning, Disability and Health, *EQ-5D* EuroQol Quality of Life Scale

### Additional questionnaires developed

The first questionnaire, to be completed on admission, comprised five sections. Section one covered personal demographic factors (gender, ethnic group, marital status) and medical information such as diagnosis of health condition, cause/nature of disability, and presence of co-morbidities. Section two described the socio-economic status of the participants, i.e. income, the participants’ monthly income, number of members in household, source of income and highest educational level. Section three described information related to the participants’ primary residence and the availability of basic needs that include running water, electricity, telephone and ablution facilities, while section four described information related to availability of transport for attending rehabilitation services. The last section related to involvement in prior research.

The questionnaire completed at discharge comprised three sections. The first section covered participants’ awareness about their rights to rehabilitation and their participation in social roles and community life activities. Section two described information about the assistive devices received and the participants’ level of satisfaction with these appliances, and section 3 described the services and interventions patients received during rehabilitation.

### Sample size

This study was largely descriptive in nature, with the focus on determining organisational capacity to deliver relevant services and the measurement of outcomes following rehabilitation. Since we selected commonly used standardised measures – specific to the selected health conditions –in the study, our sample size was determined by using the formula specific to a one-group confidence interval for means. Therefore, with confidence limits set at 95% and a desired total width of 5 for the confidence limits based on the mean score, and a standard deviation of 10 for the outcome variables (functional abilities, participation and quality of life), it was necessary to recruit at least 61 participants per site in order to detect a sufficiently powered mean value for the respective populations per site.

## Discussion

This is a groundbreaking methodological exploration as it has managed to combine instruments that are pointing to both evidence and human rights. To classify rehabilitation as an organizational structure by using Kaplan’s conceptual framework of an organizational capacity, it managed to offer the evaluation process and measurement of clients' outcomes. It is important to clarify that thought the ICF is an important outcome measure to evaluate rehabilitation services, the implementation of this tool often requires the use of other sensitive outcome measures in evaluating activity limitation and participation restrictions in clients with impairments. Having designed both the study methods, accompanied by instruments, we believe that we now have a methodology to measure rehabilitation services at both in-patient and out-patient levels of rehabilitation services. This approach using a realistic evaluation philosophy also make this methodology adaptable to any setting thereby allowing researchers the ability to use this approach in numerous studies.

## Trial status

At the time of manuscript submission the trial is in an ongoing phase of data management and analysis.

## Abbreviations

CBR: Community Based Rehabilitation
CMO: Context, Mechanisms and Outcomes
ICF: International Classification of Functioning, Disability and Health
NRP: National Rehabilitation Policy
SANPAD: South Africa Netherlands Research Programme on Alternative development
UNCRPD: United Nation Convention on the Rights of Persons with Disabilities
WHO: World Health Organisation
